# Comparative Characterization Study of a LaBr_3_(Ce) Scintillation Crystal in Two Surface Wrapping Scenarios: Absorptive and Reflective

**DOI:** 10.3389/fonc.2015.00270

**Published:** 2015-12-07

**Authors:** Saad Aldawood, Ines Castelhano, Roman Gernhäuser, Hugh Van Der Kolff, Christian Lang, Silvia Liprandi, Rudolf Lutter, Ludwig Maier, Tim Marinšek, Dennis R. Schaart, Katia Parodi, Peter G. Thirolf

**Affiliations:** ^1^Faculty of Physics, Ludwig-Maximilians-University Munich, Munich, Germany; ^2^Department of Physics and Astronomy, King Saud University, Riyadh, Saudi Arabia; ^3^Faculty of Science, University of Lisbon, Lisbon, Portugal; ^4^Physik Department E12, Technical University Munich, Garching, Germany; ^5^Faculty of Applied Science, Radiation Science and Technology, Delft University of Technology, Delft, Netherlands

**Keywords:** LaBr_3_:Ce scintillator, γ spectroscopy, medical imaging, crystal surface coating, Compton camera

## Abstract

The properties of a 50 mm × 50 mm × 30 mm monolithic LaBr_3_:Ce scintillator crystal coupled to a position-sensitive multi-anode photomultiplier (PMT, Hamamatsu H9500), representing the absorbing detector of a Compton camera under study for online ion (proton) beam range verification in hadron therapy, was evaluated in combination with either absorptive or reflective crystal surface coating. This study covered an assessment of the energy and position-dependent energy resolution, exhibiting a factor of 2.5–3.5 improvement for the reflectively wrapped crystal at 662 keV. The spatial dependency was investigated using a collimated ^137^Cs source, showing a steep degradation of the energy resolution at the edges and corners of the absorptively wrapped crystal. Furthermore, the time resolution was determined to be 273 ps (FWHM) and 536 ps (FWHM) with reflective and absorptive coating, respectively, using a ^60^Co source. In contrast, the light spread function (LSF) of the light amplitude distribution on the PMT segments improved for the absorptively wrapped detector. Both wrapping modalities showed almost no differences in the energy-dependent photopeak detection efficiency.

## 1 Introduction

Particle therapy has opened a new horizon particularly for the treatment of tumors in the vicinity of critical organs at risk, due to the sharp dose localization in the Bragg peak. However, in order to fully exploit the beneficial properties of the well localized dose deposition in the tumor volume, a precise monitoring of the ion beam range is mandatory. For this purpose, an online monitoring system based on a Compton camera designed to detect prompt (multi-MeV) γ rays, induced by nuclear reactions between the ion beam and biological tissue, is being developed at LMU Munich (7, 19). This camera is composed of six customized double-sided Si-strip detectors (DSSSD), with an active area of a 50 mm × 50 mm, a thickness of 500 μm and segmentation of 128 strips on each side, acting as scatterer (tracker), while the absorber detector is formed by 50 mm × 50 mm× 30 mm monolithic LaBr_3_:Ce scintillator. Besides the ability of detecting the scattered photon, this camera is also able to track the Compton electron (from multi-MeV prompt photons), due to the layered structure of the scatterer detectors. This feature does not only contribute to increase the reconstruction efficiency of the camera (enabling the reconstruction of incompletely absorbed photon events), but it also enhances the sensitivity to the source position of an incident photon from a Compton cone to an arc segment (5, 19).

The favorable properties of the LaBr_3_:Ce scintillator material make it the preferable detector in particular for our application in medical imaging. It has a very high light yield [61000 photons/MeV (21)] with a minor non-linearity of 6% between 60 and 1275 keV (4, 17). The material density [5.06 g/cm^3^ (4)] and effective atomic number [Z*_eff_* = 46.9 (10)], result in a high stopping efficiency. Moreover, this detector provides an excellent energy resolution from low photon energies [~3% at 662 keV (21)] up to high energies, thus keeping the ability to resolve the full energy peak from escape peaks over a wide energy range up to about 25 MeV (3). The superior timing properties of LaBr_3_:Ce, due to the fast decay time of 16 ns (1) are reflected in typical time resolutions of a few hundred picoseconds (depending on the crystal dimensions). This facilitates the use of the time-of-flight (TOF) technique, e.g., to suppress neutron background or to improve the image quality as it has been reported in positron emission tomography (PET) (6, 16).

This work aims to characterize a 50 mm × 50 mm × 30 mm monolithic LaBr_3_:5%Ce (15) scintillator crystal, wrapped with either absorptive or (after modification by the manufacturer) reflective layer, in order to determine the optimum performance of a detector configuration to be used as an absorbing detector in a Compton camera which is presently under development for proton (ion) beam range monitoring.

## 2 Materials and Methods

The monolithic LaBr_3_:Ce scintillator (50 mm × 50 mm × 30 mm) is read out by a position-sensitive (16 × 16) multi-anode photomultiplier (PMT, Hamamatsu H9500), with 256 segments of 3 mm × 3 mm each and a coupling window of 1.5 mm thickness. The crystal was encapsulated together with the PMT in an aluminum housing, which has an entrance window of 0.5 mm thickness. The light guide (specification details are unpublished) is optically coupled between the crystal and the PMT by the manufacturer. The operational voltage of this PMT was set to be −1100 V. In order to reduce the complexity of the signal processing electronics, 4 neighboring segments with an area of 6 mm × 6 mm were combined to form 64 output channels. The detector properties, such as energy resolution, photopeak detection efficiency, time resolution, and light spread function (LSF), were evaluated.

The energy resolution was studied as a function of the γ ray energy using ^152^Eu (110 kBq), ^60^Co (32 kBq), and ^137^Cs (163 kBq) calibration sources, placed in an axial distance of 25 cm of the detector surface. The data was fitted using a two-parameter function expressed as

(1) $$\frac{\Delta E}{E}=100\times \frac{\sqrt{A+B\times E}}{E}$$

where A and B are free parameters (14). In addition, the position-dependent energy resolution was investigated by scanning the detector with a 1 mm collimated ^137^Cs source of 86 MBq activity and a 2 dimensional step size of 6 mm, forming 8 × 8 irradiation positions with the pencil γ ray beam pointing to the center of the respect PMT pixel group and 5 min measurement time at each position. At each irradiation position, the relative energy resolution $\frac{△E}{E}$ was determined, thus generating an energy resolution map for the detector crystal with reflective and absorptive side coating, respectively. In both measurements, the evaluation of the energy resolution was based on the sum dynode signal of the PMT (Hamamatsu H9500). This signal was fed to an amplifier and Constant Fraction Discriminator (CFD) module (Mesytec, MCFD-16) and then to a VME-based Charge-to-Digital Convertor (Mesytec, MQDC-32) to enable digitized list-mode data acquisition and subsequent spectra analysis.

The photopeak detection efficiency of the LaBr_3_:Ce detector was evaluated using the known activities of the calibration sources. This required measuring the ratio of photons detected in the photopeak to the number of initially emitted γ rays for the specific transitions. In this case, a ^152^Eu source of 110 kBq activity was used in order to cover a wide range of photon energies between 121 and 1408 keV. The energy spectrum was derived from the sum dynode of the PMT. The data was corrected by background subtraction. Dead time and solid angle corrections were applied.

The timing performance of the LaBr_3_:Ce scintillator was investigated relative to a fast reference plastic detector (BC-418) using a coincidence method. First, the time resolution of the reference detector was determined by measuring the coincidence time between two simultaneously emitted γ rays from a ^60^Co source, using two identical plastic detectors (BC-418) coupled to fast PMTs (photonis XP2020/Q). The two signals of these detectors were fed to an amplifier plus CFD module (Mesytec, MCFD-16) and subsequently to a Time-to-Digital Converter (TDC, C.A.E.N. Mod. V775). Then, the time resolution of one reference detector Δ*T_plast._*_1_ was extracted according to

(2) $$\Delta T_{\mathrm{plast}.1}=\sqrt{\frac{{\left(△t_{\mathrm{plast}.1+2}\right)}^{2}}{2}}$$

where the Δ*T_plast._*_1+2_ is the total time resolution measured by the two identical reference detectors.

Subsequently, one of the reference detectors was replaced by the LaBr_3_:Ce detector in order to measure the coincidence time resolution of this system. Knowing the time resolution of the reference detector and the combined time resolution (Δ*T_tot_*) of plastic and LaBr_3_:Ce scintillator, the time resolution of the LaBr_3_:Ce detector can be obtained as

(3) $$\Delta T_{\mathrm{LaB}r_{3}}=\sqrt{{\left(\Delta T_{\mathrm{tot}}\right)}^{2}-{\left(\Delta T_{\mathrm{plast}.1}\right)}^{2}}$$

Finally, the spatial resolution properties of the LaBr_3_:Ce scintillator was evaluated by the Light Spread Function (LSF), defined as the FWHM of the radial projection of the light distribution of the multi-anode PMT pixels. In order to extract the relevant light amplitude distributions correlated to the incident γ rays, some correction steps have to be applied consecutively:

- Gain matching: Since each channel of the 64 PMT output signals was processed by individual spectroscopic electronics, potential gain variations between channels have to be corrected. Therefore, two pulser signals with different amplitudes (50 and 100 mV) were injected to each amplifier channel in order to match the relative amplification gains of all channels.
- QDC pedestal: The charge-to-digital converter (QDC) continuously produces low-amplitude signals originating from the dark current. The intensity and energetic position of this signal varies from channel to channel. Thus, digitized data was acquired without input signal to the QDC in order to define (after applying a Gaussian fit to the pedestal peaks) a fixed pedestal subtraction threshold that was determined as 3 σ above the pedestal peak centroid.
- PMT uniformity: In order to allow for a correction of the gain variations between the 256 pixels of the multi-anode PMT (H9500), a gain non-uniformity matrix is provided by the PMT manufacturer (Hamamatsu). Since each 4 neighboring pixels of the PMT were electronically summed in this study, the corresponding gain values of the non-uniformity matrix were averaged to derive new correction factors for the 64 output channels. These relative correction factors range from 1.0 to 1.8.
- Crystal light distribution uniformity: Scattering or reflections of the scintillation light mainly in the corner or edge regions of the crystal could lead to an inhomogeneous spatial response of the crystal to impinging radiation. This can be corrected by registering the light amplitude distribution, resulting from a homogeneous flood source covering the crystal front surface, allowing to derive a correction map. Fortunately, the LaBr_3_:Ce crystal offers an elegant alternative. Since the internal radioactivity of the LaBr_3_:Ce detector (here 140 Bq, exhibiting besides distinct transitions from ^138^La and ^227^Ac (11, 18) a continuum ranging up to about 2.6 MeV) can be assumed to be homogeneously distributed inside the crystal with isotropic emission of the corresponding γ rays. The photon energy region of this background radiation equivalent to the impinging γ rays (in this case 662 keV from a ^137^Cs source) was used to determine the position-dependent correction matrix. This takes into account the energy dependence of the scatter/reflection processes near corners and edges of the crystal, affecting the light collection behavior.

After applying the above corrections, an 8 × 8 grid scan of the LaBr_3_:Ce detector using a 1-mm collimated ^137^Cs (86 MBq) source was performed to visualize the correlated movement of the source position. Then, one of the four central irradiation position measurements from this grid scan was selected to derive the LSF by performing a radial projection of the light amplitude distribution of the PMT pixels.

## 3 Results and Discussion

### 3.1 Energy Resolution

**Figure 1:**
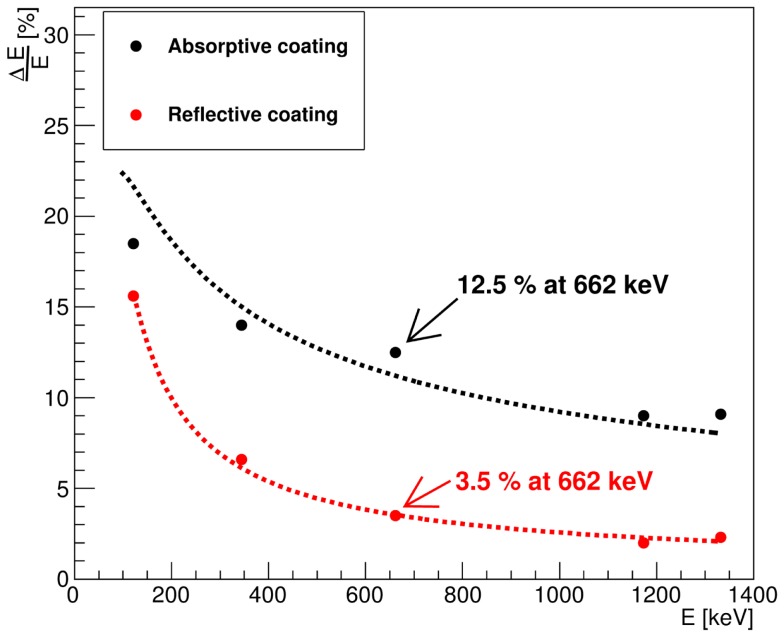
**Energy resolution as a function of the photon energy values measured by a LaBr_3_:Ce detector with reflective (red) and absorptive (black) side surface coating obtained with ^152^Eu, ^137^Cs, and ^60^Co calibration sources**. The dotted lines represent a two-parameter function fit as indicated in Equation (1).

Figure 1 displays the energy resolution for photon energies from 121 to 1332 keV, as determined for the reflectively and absorptively coated LaBr_3_:Ce crystal, respectively. The dotted curves parameterize the energy dependence of the relative energy resolution according to the two-parameter function indicated in Equation (1). The relative energy resolution $\frac{\Delta E}{E}$ was found to be 12.5 and 3.5% at 662 keV for the absorptively and reflectively wrapped crystal, respectively. Throughout the energy range, the reflectively wrapped crystal exhibited a significantly improved energy resolution. In general, the energy resolution ΔE/E of a scintillation detector read out by a photomultiplier can be expressed as

(4) $${\left(\frac{\Delta E}{E}\right)}^{2}=\delta_{\mathrm{intr}}^{2}+\delta_{\mathrm{tran}}^{2}+\delta_{\mathrm{st}}^{2}$$

where *δ_intr_* is the intrinsic detector resolution affected, e.g., by crystal inhomogeneities, *δ_tran_* is the transfer resolution that is correlated to the optical coupling properties of the crystal to the PMT readout, including the photocathode quantum efficiency as well as the focusing of photoelectrons to the first dynode, and *δ_st_* is the statistical contribution of the PMT (12). The last two factors will determine the statistical uncertainty of the PMT, as it is directly affected by the number of photoelectrons generated at the photocathode and the photoelectron collection efficiency at the first dynode (13).

**Figure 2:**
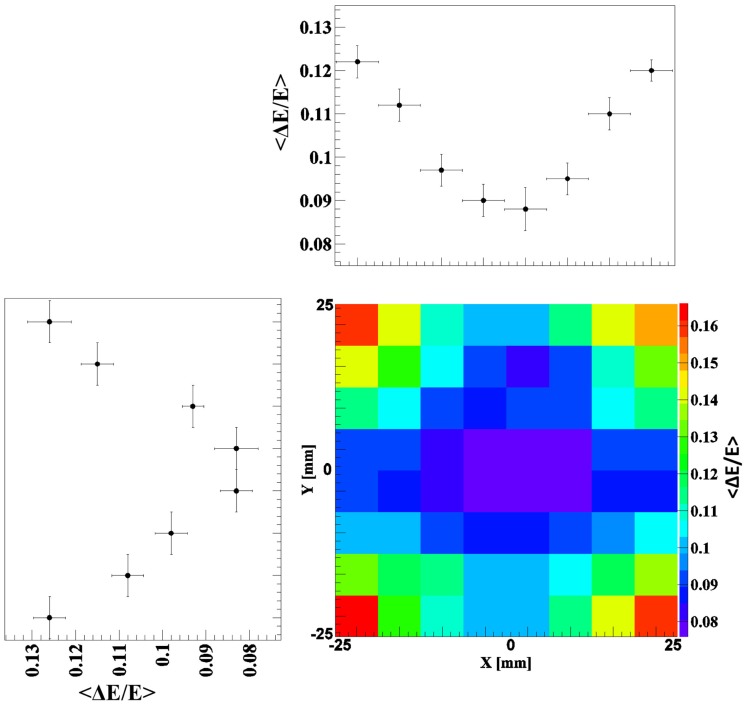
**2D energy resolution map together with its X and Y projection obtained by scanning the absorptively wrapped LaBr_3_:Ce crystal with a 1-mm collimated ^137^Cs source and step a size of 6 mm in x and y direction**.

**Figure 3:**
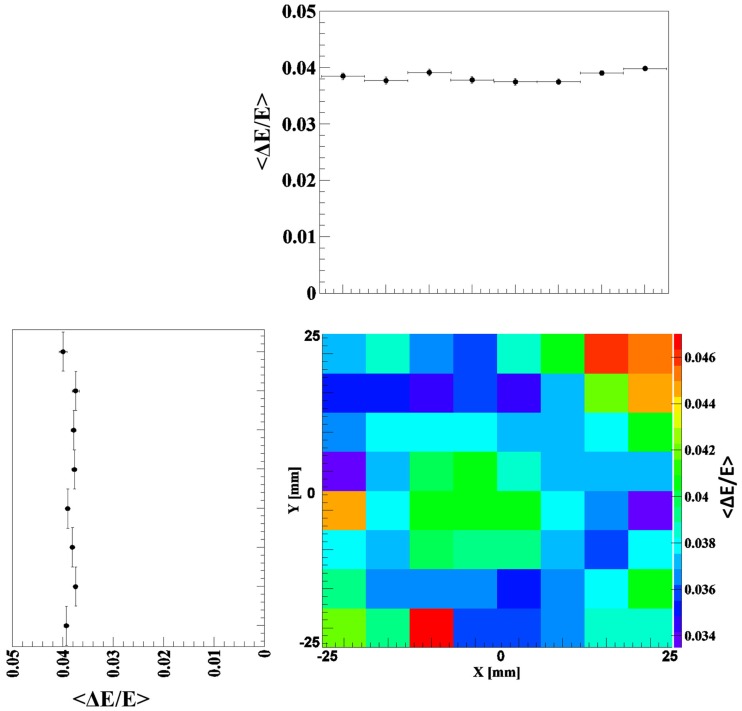
**2D energy resolution map measured by scanning the reflectively coated LaBr_3_:Ce crystal with a 1-mm collimated ^137^Cs source and step a size of 6 mm in x and y direction**. The x and y projections (averaged over the complementary direction) show an almost position independent energy resolution of 3.8% on average.

Since the same crystal and optical coupling were used with reflective and absorptive crystal wrapping, the intrinsic term can safely be expected to give the same contributions to the overall energy resolution in both scenarios. As the generated scintillation light is partially absorbed by the absorbing wrapping material and consequently the number of the photoelectrons that reach the PMT is drastically reduced, the statistical term *δ_st_* should contribute to the degradation of the energy resolution in the absorptively coated crystal, since *δ_st_* is inversely proportional to the square root of the number of photoelectrons.

(5) $$\delta_{\mathrm{st}}=2.35\sqrt{\frac{1+\nu_{M}}{N_{\mathrm{phe}}}}$$

where *v_M_* is the variance of the PMT gain, typically between 0.1 and 0.2, and *N_phe_* is the number of photoelectrons (12). The transfer term *δ_tran_* is also expected to contribute to the energy resolution deterioration in the absorptively wrapped crystal, since in this crystal the collection of the scintillation light at the photocathode strongly depends on the interaction position at which the scintillation light is generated. This can be noticed throughout the study of the spatial dependence of the energy resolution using the 1 mm collimated ^137^Cs source. Moreover, even for a given position of interaction, the probability for a scintillation photon to arrive at the photocathode will depend much more strongly on the initial angle of emission than in the reflectively wrapped crystal. Figure 2 shows the resulting 2D energy resolution map of the absorptively wrapped LaBr_3_:Ce crystal at 662 keV. The energy resolution is gradually degrading from 8% in the central region to about 10 and 16% at the detector’s edges and corners, respectively. This can be attributed to the reduction of scintillation light reaching the PMT (thus reducing the number of photoelectrons) due to the absorption of scattered and reflected photons hitting the absorptively coated side surfaces of the scintillation crystal. This effect is much stronger for scintillation photons generated in the edge or corner regions compared to the central region of the crystal. In contrast, this effect disappears with the reflective coating of the LaBr_3_:Ce crystal as indicated in Figure 3. The corresponding 2D energy resolution is only slightly varying with the irradiation position, as can be seen from the respective x and y projections (averaged over the complementary dimension). An averaged relative energy resolution $\frac{\Delta E}{E}=3.8\pm 0.04\%$ is achieved in this scenario. The drastic improvement by about a factor of 2.5–3.5 compared to the absorptive coating clearly emphasizes the need to preserve the full amount of scintillation light (and thus photoelectrons *N_phe_*) via the reflective wrapping of the crystal, thus, reducing the statistical fluctuations in of *N_phe_* at each irradiation position.

### 3.2 Photopeak Efficiency

**Figure 4:**
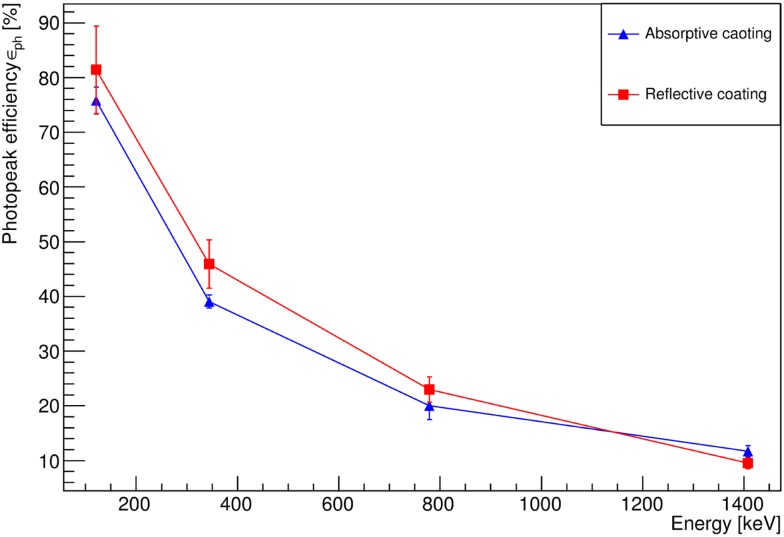
**Photopeak detection efficiency of the absorptively (blue) and reflectively (red) wrapped LaBr_3_:Ce crystal using a ^152^Eu calibration source**. Background, solid angle, and DAQ dead time corrections were applied to these data. The solid lines are to guide the eye.

The LaBr_3_:Ce photopeak detection efficiency *ε_ph_* was determined with reflective and absorptive crystal coating over an energy range between 121 and 1408 keV, as displayed in Figure 4. With both types of crystal surface coating, the full energy detection efficiency, corrected for solid angle and data aquisition dead time, starts from high values of *ε_ph_* ≈ 80% at low energies (121 keV) due to the crystal thickness of 30 mm, the high effective atomic number Z*_eff_* and the density of the detector material, rendering the probability of the photoelectric interaction to be dominant in this energy region. However, the observed drop of *ε_ph_* with increasing photon energy correlates with the emerging dominance of multiple interactions, such as Compton scattering for high energy photons contributing to reduce the photopeak efficiency. Figure 4 also shows that the photopeak detection efficiency within experimental uncertainties is almost independent of the different surface coatings as expected.

### 3.3 Time Resolution

**Figure 5:**
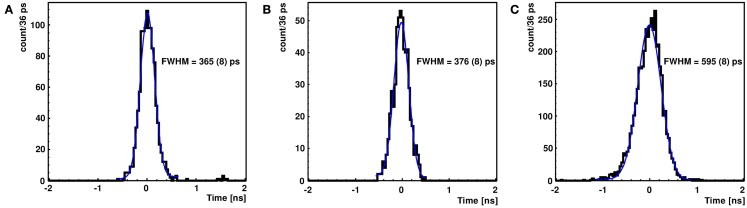
**Time coincidence peak of two simultaneously emitted photons from ^60^Co measured by two identical plastic detectors (BC418) (A) and by a reflectively (B) as well as an absorptively (C) wrapped LaBr_3_:Ce detector measured against the reference plastic detector**. The blue curve represents a Gaussian fit used to derive the indicated FWHM values.

The timing properties of the LaBr_3_:Ce detector were investigated for the alternative crystal coatings relative to a fast reference plastic scintillator. Figure 5A shows the coincidence time peak of two simultaneously emitted photons from ^60^Co, measured by two identical plastic detectors (BC-418), exhibiting a FWHM of 365 ± 8 ps. From Equation (2), the time resolution of a single reference detector was found to be 258 ± 5 ps (FWHM). A similar result was obtained for a BC-418 scintillation detector coupled to the same PMT type (photonis XP2020/Q) by (6) to be 235 ps (FWHM). Figures 5B,C indicate 376 ± 8 ps (FWHM) and 595 ± 8 ps (FWHM) as the coincidence time measured for the reflectively and absorptively wrapped LaBr_3_:Ce detector, respectively, in coincidence with the reference plastic detector. Using the measured time resolution of the reference detector, the time resolution of the LaBr_3_:Ce detector was extracted using Equation (3) to be 536 ± 6 ps (FWHM) with absorptive and 273 ± 6 ps (FWHM) with reflective wrapping. Since the same crystal, PMT, electronics and time pick-off method were used in both side surface wrapping scenarios, the improvement in the time resolution of the LaBr_3_:Ce detector by more than a factor of 2 can clearly be attributed to the maximized light collection in the reflectively wrapped crystal. Consequently, the number of collected photoelectrons per event is correspondingly maximized, thus reducing the statistical fluctuations that affect the time resolution, which scales inversely proportional to the square root of the number of photoelectrons (2, 8).

### 3.4 Light Spread Function

**Figure 6:**
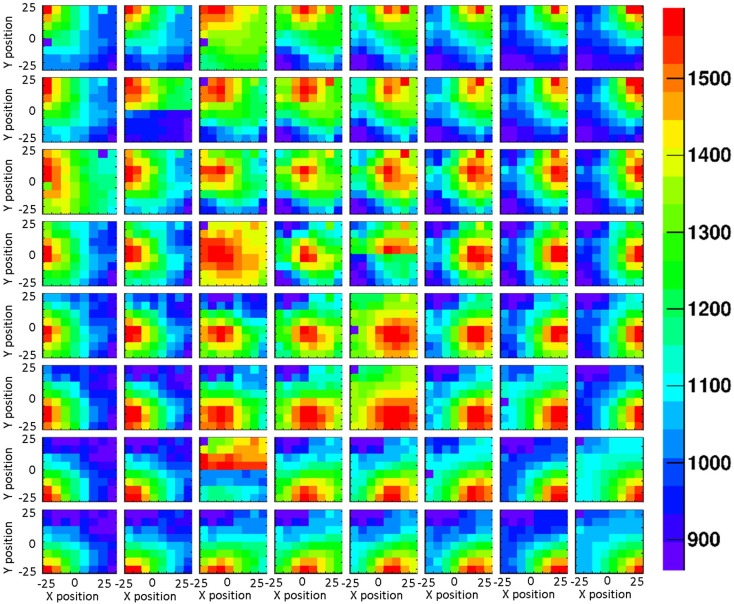
**2D light amplitude distribution obtained from an 8 × 8 grid scan of the absorptively wrapped LaBr_3_:Ce detector, using a 1 mm collimated ^137^Cs source with a step size of 6 mm in x and y direction**. Each subpicture represents light amplitude distribution of an irradiation position. The corresponding correlation of the resulting light amplitude distributions (after the analysis steps described in Sect. 2) with the shift of the irradiation position is clearly visible.

**Figure 7:**
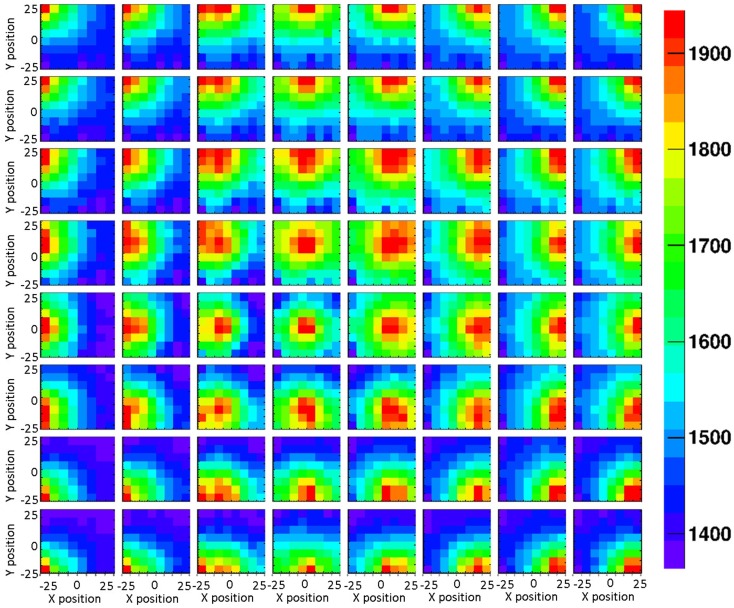
**A grid scan of 8 × 8 irradiation positions of the reflectively coated LaBr_3_:Ce detector, using a 1-mm collimated ^137^Cs source**. The source irradiation position can be clearly tracked by the intensity of the detector 2D light distribution.

The impact of the crystal surface coating on the position sensitivity of the LaBr_3_:Ce detector was studied using the 1 mm collimated ^137^Cs source. Figures 6 and 7 show 2D light amplitude distributions for each irradiation position on a 8 × 8 grid with 6 mm step size in x and y direction. The irradiation position is clearly correlated with the shape of the measured light distribution both with absorptive and reflective surface wrapping after applying the correction steps discussed in section 2.

**Figure 8:**
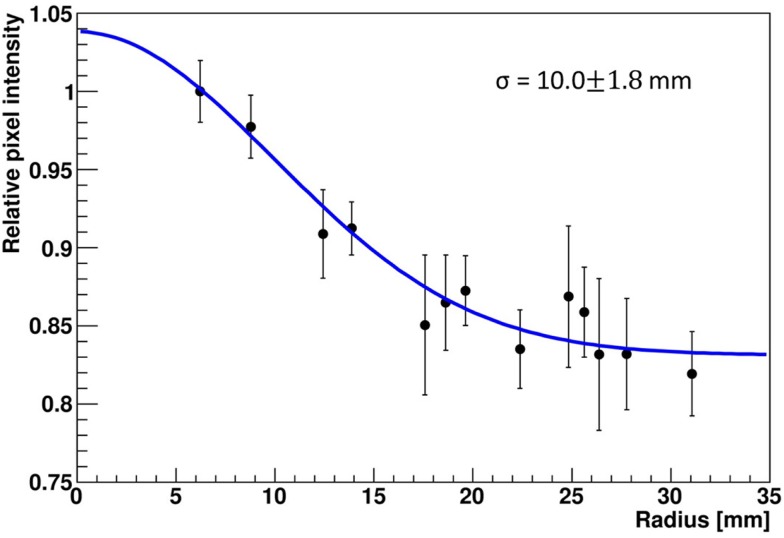
**Radial projection of the 2D light amplitude distribution obtained from a central irradiation position of the absorptively wrapped LaBr_3_:Ce detector**. In this study, 4 neighboring segments of the 16 × 16 multi-anode PMT were combined. The blue curve represents a Gaussian fit to the data, the resulting width σ is indicated.

**Figure 9:**
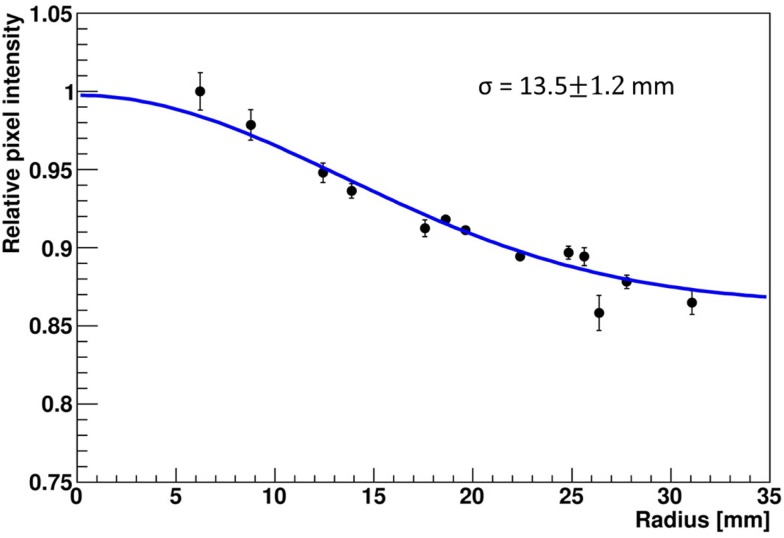
**Radial projection of the 2D light amplitude distribution derived from a central irradiation position of the reflectively wrapped LaBr_3_:Ce detector**. The blue curve represents a Gaussian fit to the data, the resulting width σ is indicated.

**Figure 10:**
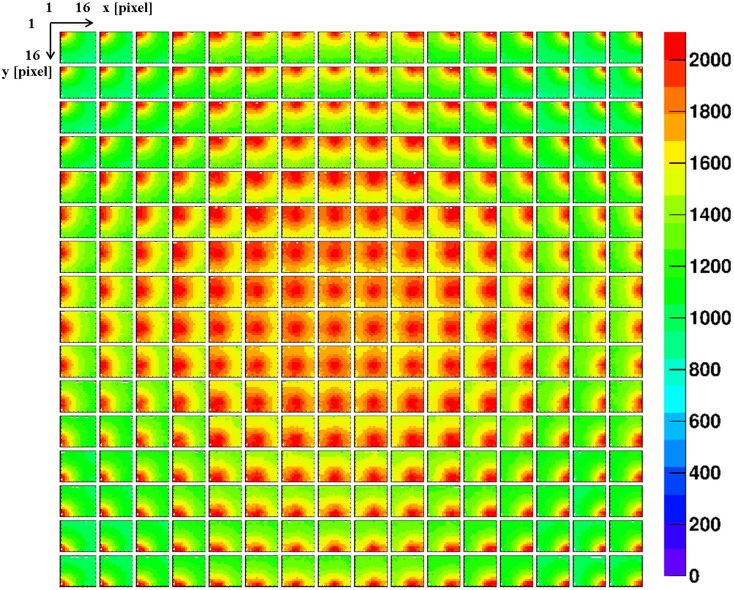
**A grid scan of 16 × 16 irradiation positions of the reflectively coated monolithic LaBr_3_:Ce detector, using a 1 mm collimated ^137^Cs source and a grid step size of 3 mm in x and y direction**. All 256 segments of the multi-anode PMT are individually read out. The resulting 2D light amplitude distribution of each irradiation position clearly indicates a systematically different pattern for the 256 different source positions.

**Figure 11:**
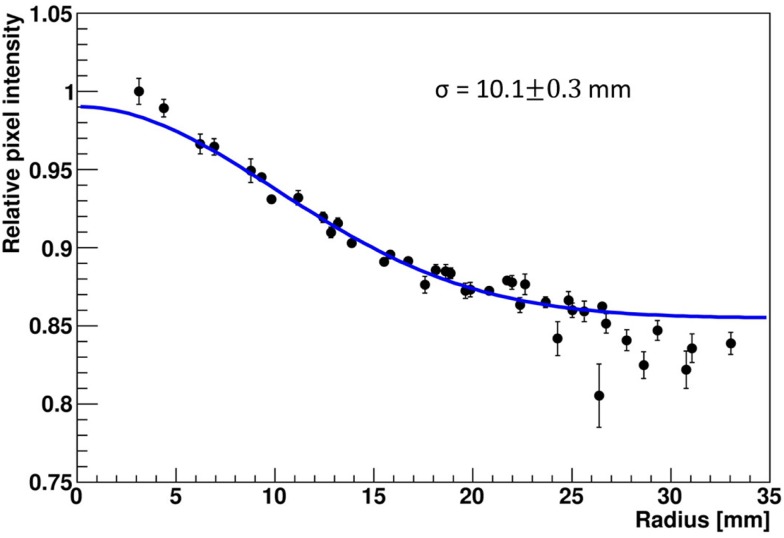
**Radial projection of the 2D light amplitude distribution derived from a central irradiation position of the reflectively wrapped LaBr_3_:Ce detector coupled to a 16 × 16 multi-anode PMT**. The blue curve represents a Gaussian fit to the data, the resulting width σ is indicated.

The LSF, which corresponds to a radial projection of the 2D light amplitude distribution, is used to evaluate the impact of the crystal wrapping type on the detector’s spatial resolution. The absorptively wrapped LaBr_3_:Ce detector exhibits a LSF of 23.5 ± 4 mm FWHM (σ = 10.0 ± 1.8 mm) derived from the radial projection fit of the 2D light distribution as indicated in Figure 8. In contrast and derived from Figure 9, it was measured to be 31.7 ± 3 mm FWHM (σ = 13.5 ± 1.2 mm) for the reflectively LaBr_3_:Ce detector. As expected, the reflective coating degrades the position sensitivity of the detector due to the scintillation light scattering at the edges and corners of the crystal. However, this degradation does not prevent the detector from resolving the photon source-position correlation as shown in Figure 7. While so far, measurement and analysis of the LaBr_3_:Ce has been performed using an initial version of the signal processing electronics with 64 signal readout channels, in the further progress of the R&D project the readout electronics was upgraded to the full capacity of 256 channels needed for an individual readout of the 16 × 16 multi-anode PMT segments. Consequently, the position-dependent collimated irradiation was repeated with a finer grid step of 3 mm in x and y direction, resulting in the light amplitude correlation map shown in Figure 10, where 16 × 16 2D maps are displayed, each with 16 pixel in x and y, respectively (compared to 8 × 8 pixel in Figures 6 and 7). The higher granularity of the segmented readout and the scan stepsize enables as well a refined analysis of the LSF for the reflectively coated crystal (note that the electronics upgrade was performed after the crystal modification from absorptive to reflective coating). The resulting LSF is shown in Figure 11 exhibiting a width of 23.7 ± 0.7 mm FWHM (σ = 10.1 ± 0.3 mm), comparable to the findings for the less segmented, absorptively wrapped crystal. Based on these findings, the position information for the impinging primary photon is planned to be derived from the monolithic LaBr_3_:Ce scintillator using the “k-nearest neighbor” (k-NN) method developed at TU Delft (20), which requires an even finer 2D grid scan of the detector (0.5 mm collimation, 0.5 mm stepsize) (9).

## 4 Conclusion

A monolithic LaBr_3_:Ce detector (50 mm × 50 mm × 30 mm) coupled to a position-sensitive multi-anode PMT was characterized with reflective and absorptive crystal surface coating for the purpose of optimizing the absorbing detector of a Compton camera, intended to be used as a monitoring system for ion (proton) beam range monitoring in hadron therapy. The photopeak efficiency of the detector is negligibly affected by the type of crystal coating. The reflective coating contributes to improving the energy and time resolution of the detector, because it enhances the light collection that reduces statistical fluctuations in both cases. However, this type of coating degrades the detector position sensitivity due to the increase in light scattering at the edges and corners of the crystal. While at the first glance, it appears counterproductive to use reflective side surface wrapping (plus polished crystal surface treatment) in a scenario where position resolution is targeted via a multi-anode PMT readout, in our case it is nevertheless mandatory, since an optimized energy resolution is of equal importance when operating the crystal in the context of a Compton camera. From the results obtained in this study, the reflectively wrapped LaBr_3_:Ce scintillator qualifies to be the optimum choice for the Compton camera absorbing detector. This is further emphasized by the presented measurements with the upgraded, highly granular electronic readout for all of the 256 PMT segments.

## Author Contributions

SA: main author, acquisition, data collection, analysis, interpretation; IC, HK, CL, SL, TM: participate in experimental work and design; RG: experimental design, revising, and final approval to be published; RL: data acquisition; LM: experimental design; DS, KP, PT: interpretation, revising, and final approval to be published.

## Conflict of Interest Statement

The authors declare that the research was conducted in the absence of any commercial or financial relationships that could be construed as a potential conflict of interest.
